# Supplementary figures and images for: Laser Pulses for Studying Photoactive Spin Centers with EPR
Source: Micromachines (Basel). 2025 Mar 28;16(4):396. doi: 10.3390/mi16040396 (PMC12029438; doi:10.3390/mi16040396)

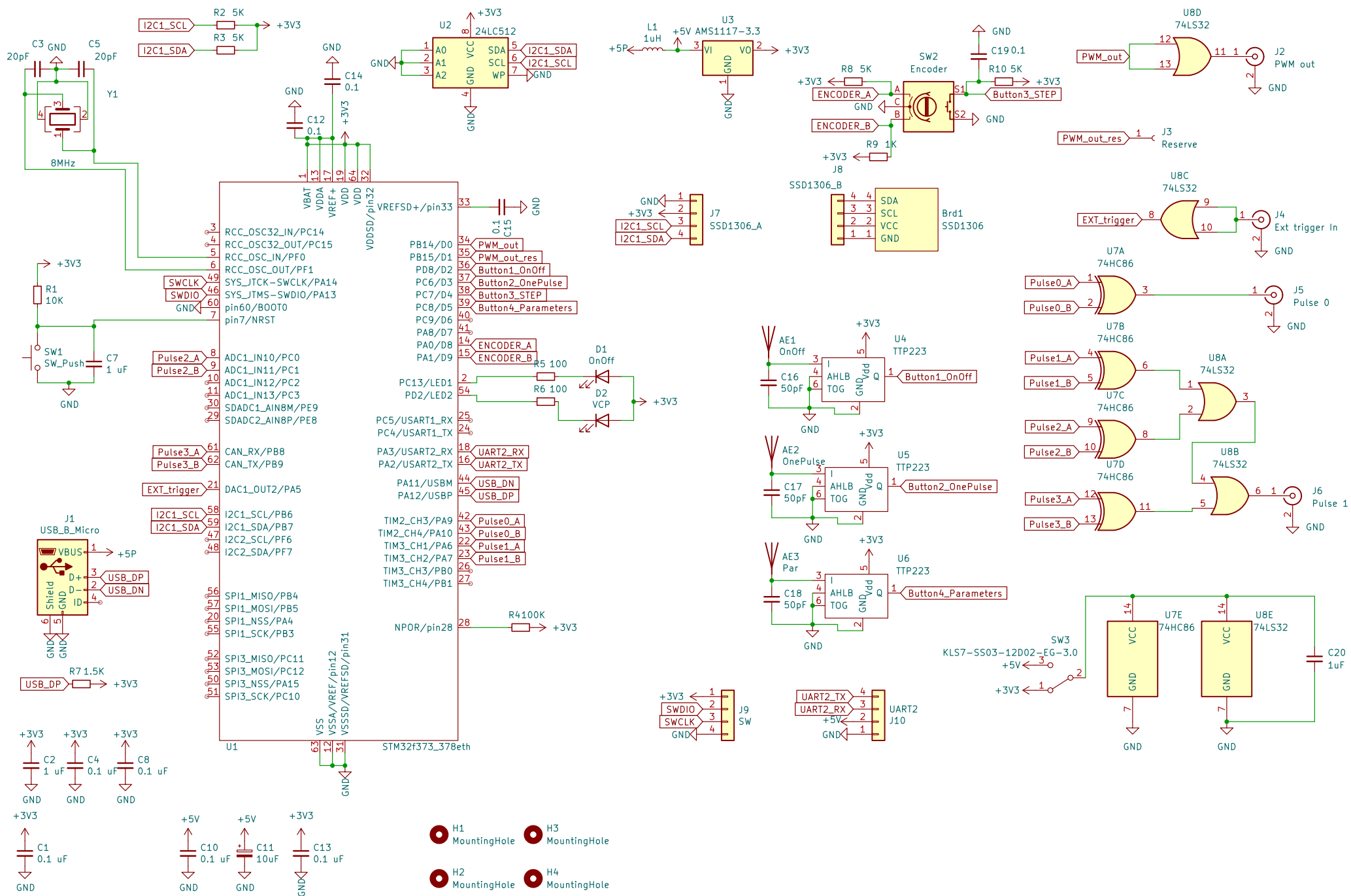

Supplement: Supplementary file 1 [file micromachines-16-00396-s001.zip › micromachines-3497941-supplementary.pdf]
